# Heavy Metal Assessments of Soil Samples from a High Natural Background Radiation Area, Indonesia

**DOI:** 10.3390/toxics10010039

**Published:** 2022-01-15

**Authors:** Eka Djatnika Nugraha, June Mellawati, Wahyudi, Chutima Kranrod, Makhsun, Hirofumi Tazoe, Haeranah Ahmad, Masahiro Hosoda, Naofumi Akata, Shinji Tokonami

**Affiliations:** 1 Research Centre for Technology of Radiation Safety and Metrology, The National Research and Innovation Agency of Indonesia (OR TN-BRIN), Jakarta 12440, Indonesia; eka.dj.n@batan.go.id (E.D.N.); june_mellawati@batan.go.id (J.M.); wah_yudi@batan.go.id (W.); makhsun@batan.go.id (M.); 2 Department of Radiation Science, Graduate School of Health Sciences, Hirosaki University, Hirosaki 036-8560, Japan; m_hosoda@hirosaki-u.ac.jp; 3 Department of International Cooperation and Collaborative Research, Institute of Radiation Emergency Medicine, Hirosaki University, Hirosaki 036-8560, Japan; kranrodc@hirosaki-u.ac.jp (C.K.); tazoe@hirosaki-u.ac.jp (H.T.); 4 Department of Environmental Health, Health Polytechnic of Mamuju, Mamuju 91511, Indonesia; haeranahahmad@poltekkesmamuju.ac.id; 5 Department of Radiation Measurement and Physical Dosimetry, Institute of Radiation Emergency Medicine, Hirosaki University, Hirosaki 036-8560, Japan; 6 Department of Radiochemistry and Radioecology, Institute of Radiation Emergency Medicine, Hirosaki University, Hirosaki 036-8560, Japan; akata@hirosaki-u.ac.jp

**Keywords:** toxic, lead, natural radiation

## Abstract

Mamuju, Indonesia, is an area with high natural background radiation. This study assesses heavy metal content in soil samples from this area to determine the level of public and environmental hazard it presents. This study analyzes natural radionuclide elements using high purity germanium (HPGe) gamma spectrometry and performs heavy metals analysis using a flame atomic absorption spectrometry (FAAS). Moreover, pollution indices and descriptive analyses were used to assess heavy metal contamination in the environment and the correlation between heavy metals and radionuclides. The results demonstrate that soil samples in several areas of Mamuju contain a high concentration of the natural radionuclides ^226^Ra and ^232^Th, and that heavy metal concentrations in the soil decrease in the sequence Zn > Pb > Cr > Cu > Ni > Cd. This study revealed that soil samples from Mamuju are moderately contaminated. There was a strong positive relationship between ^226^Ra, ^232^Th, ambient dose equivalent rate, and Pb. Ecological risk index (RI) and cumulative pollution index (IPI) values in Mamuju are 2.05 and 125, respectively, which are possible hazards to human health as a result. Pb concentration in the Mamuju soil samples ranged from 109 to 744 mg kg^−1^, exceeding the worldwide average of 27 mg kg^−1^.

## 1. Introduction

Heavy metals are widely distributed in the environment and are often associated with pollution, contamination, and toxicity due to non-bio-degradable and persistence in nature [1,2]. In the environment, there are both natural and anthropogenic sources of heavy metals [1]. The main natural source is geological degradation, such as rock weathering or thermal springs. The anthropogenic sources come from sewage sludge, fossil fuel combustion, industrial processes, and organic and inorganic fertilizers [3].

Recently, heavy metals in the environment have increased beyond acceptable limits through development activities, including industry and agriculture. These heavy metals can be toxic and accumulate in the soft tissues of animals, plants, and even humans when they enter the body through food, water, air, or skin. Soil is often a repository of heavy metals because soil particles, such as clay and humus, have a charge that helps metal cations bind to the soil, thereby preventing their release, even temporarily [4]. Heavy metal toxicity can cause several diseases that attack almost all vital organ functions [5]. Some heavy metals that can cause health include copper (Cu), chromium (Cr), cadmium (Cd), lead (Pb), nickel (Ni), and zinc (Zn).

Pb is a harmful environmental pollutant with high toxic effects on many body organs. Pb is absorbed from the respiratory and digestive systems. Due to immune modulation, oxidative, and inflammatory mechanisms, Pb exposure can induce neurological, respiratory, urinary, and cardiovascular disorders. Cr is found in the Earth’s crust and industrial processes. The main route of Cr exposure is via ingestion and can cause various diseases, such as renal, neurological, and several cancers, including lungs, bladder, kidneys, thyroid, testicles, and bone. Cd, although rare, occurs naturally in soil. A high concentration of Cd in the soil can occur following industrial activities, with the main exposure route being ingestion. Health effects due to this heavy metal are degenerative bone diseases, kidney failure, and lungs diseases. Zn and Ni are essential heavy metals in the human body. However, exposure to extreme elements can be harmful to respiratory diseases. Cu, as well as Cr, is found in the Earth’s crust. The health effect of this heavy metal concerns abdominal disorders and metabolic activity abnormalities [5,6,7,8].

Several studies have also discussed heavy metal pollution in areas with high levels of natural radionuclide elements in the soil, such as areas around uranium mines with high background radiation [9,10,11]. As with heavy metals, high concentrations of radionuclides in the soil in an area can cause radiation-induced diseases, such as cancer and non-cancer diseases. Uranium exploration activities employing poor residue management techniques adversely affect the environment. Significant concentrations of radionuclides in the soil and water are generally accompanied by significant heavy metal contents [12].

Mamuju, Indonesia has a relatively high average radiation dose rate due to its high natural background radiation level [13,14,15,16]. Mapping of radiation dose rates at several locations in the Mamuju area was conducted by the Research Center for Radiation and Metrology Technology (PRTKMR) of the National Research and Innovation Agency of Indonesia (OR TN-BRIN) and Hirosaki University, Japan. A geometric mean of 613 nSv h^−1^ with a range of 200 to 2300 nSv h^−1^ was found [13].

A previous study has found that Mamuju has a unique geological condition, with 15 types of rock with different mineral contents, 11 types of class C minerals, 6 types of Class B minerals, and 5 types of class A minerals found in this area [14]. Volcanic rocks with ultrapotassic affinity and andesitic basaltic types are grouped in the Adang volcanic rock area, formed on the active continental margin (ACM) with the microcontinental crust of the SW Sulawesi block [17,18]. Rosianna et al. (2020) measured radioactivity in samples of breccia rock, tephra-phonolite, phonolite, phono-tephrite, and trachyte in several areas of Mamuju using a high-purity germanium detector (HPGe) with an activity concentration of ^238^U from 539 to 128,699 Bq kg^−1^ and ^232^Th from 471 to 288,639 Bq kg^−1^ [19]. According to the mineralization, the main minerals in Mamuju are davidite and thorianite that contain heavy metals, uranium, and thorium [20]. Moreover, secondary minerals are gummite and autunite [17].

This study assesses heavy metal levels in soil samples taken in Mamuju in order to determine the possibility of environmental and public hazards resulting from heavy metals in this area. Heavy metal pollution in the soil can have harmful effects on groundwater, agricultural production, food safety, and human health because the soil is the most important ecosystem for human survival and development. Most people living in Mamuju are farmers, and their daily life in the area is, therefore, heavily dependent on nature. Most residents mainly consume locally grown food from traditional markets. Therefore, the determination of the heavy metal content in the local soil is fundamental to the identification, monitoring, and assessment of potential sources of pollution in the area [6,9].

This paper describes the first approach to assess heavy metal contamination in Mamuju. Previous studies on Mamuju examined specific areas rather than the region as a whole, and had limited scope, focusing purely on natural radioactivity, radiation dose assessment, and radioactive mineral exploration [13,14,15,19,21].

## 2. Materials and Methods

### 2.1. Study Area

Mamuju is located on the western edge of Sulawesi Island, between Mamuju Bay to the north and Labani Bay to the south. The topography of the Mamuju area consists of a western coastal area and an eastern mountainous area [14,22]. The landscape is still natural in that there has been no mining activity. At 62% of the population, the majority of Mamuju residents are farmers; the primary agricultural product is cacao, an exported commodity [13]. Due to these circumstances, we assessed several heavy metals in soil samples, such as Cu, Cr, Cd, Pb, Ni, and Zn, which might affect the health of the Mamuju residents based on their activities and geological conditions.

**Figure 1:**
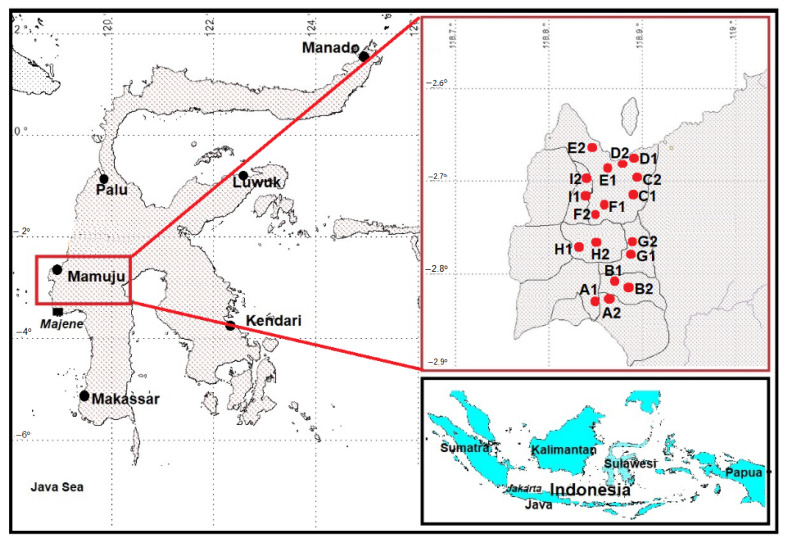
Study area. The red dots represent the sample sites used in this study, and the black dots represent the capital cities of Indonesian provinces.

Soil samples were obtained from the sub-districts of Mamaju city (Binanga Village, Tama Sapi, Mamuyu Village, So’do, Rimuku, and Karema), Tapalang sub-district (Takandeang Village, Tabanga-banga, and Palada), and Northern Botteng Village. Figure 1 shows a map of these locations.

### 2.2. Sampling and Preparation

Soil samples were collected from 18 different locations in Mamuju. Each sample consisted of 1 kg of soil extracted at a depth of 5–20 cm from the soil surface using a trowel. Then, soil samples were stored in polymer bags. In the laboratory, the extraneous plant material, rocks, gravel, and roots were removed from the soil samples, then dried in an oven at 105 °C for 24 h to remove the moisture content. The soil samples were then crushed by hand, sieved using a 50–60 mesh sieve, and separated into two parts for natural radionuclide element analysis and heavy metal analysis.

### 2.3. Identification of Heavy Metal Elements

The samples after preparation were heated in a furnace at 400 °C for 2 h to remove organic matters. An aqua-regia extraction method was then used to determine the total metal content of each sample. This method consists of the partial digestion (by HNO_3_–HCl) of 1 g of the soil sample for 2 h at 90 °C. Afterward, the sample was diluted to 100 mL with de-ionized water, left for 3 h, and then filtered [23,24]. All chemicals and standard solutions were purchased from Merck (Darmstadt, Germany). A flame atomic absorption spectrometer (ContrAA 300, Analytic, Jena, Germany; ‘FAAS’ hereafter) was used to carry out heavy metal element analysis. The FAAS was calibrated with the relevant Analytic Jena FAAS grade standards. All results were measured in triplicate, and average values were calculated. Moreover, we measured the certified reference material (CRM) OREAS 465 (Tanzania-Mantra Resources Nyota Prospect) to obtain accurate and precise measurement. Obtained values are in good agreement with the certificate of CRM. The minimum detection concentrations (MDCs) of Cu, Cr, Cd, Pb, Ni, and Zn were 0.15, 0.41, 0.14, 1.5, and 0.22 mg kg^−1^, respectively.

### 2.4. Radioactivity Measurement

A high purity germanium (HPGe) detector gamma spectrometer (Ortec, Oak Ridge, TN, USA) was used to measure the radioactivity of the sample. Each approximately 1 kg sample was weighed and sealed in a standard Marinelli container, and placed for approximately 30 days before further gamma spectrometer measurement to allow the decay products to reach secular equilibrium. The counting time was approximately 80,000 s. The peak energy values of 351 and 609 keV were used to calculate ^238^U and ^226^Ra, respectively. The energy peaks of 238, 581, and 911 keV were used for ^232^Th, and the single peak of 1460 keV was used for ^40^K. The MDCs of ^226^Ra, ^232^Th, and ^40^K used for this measurement were 0.0052, 0.0052, 0.0034, and 0.0146 Bq kg^−1^, respectively. Equation (1) is used to calculate the radioactivity concentration from these measurements:

(1) $$A=\frac{N}{EYWf_{c}}$$

where *E* is counting efficiency, *Y* is energy yield, *N* is net count per second, *W* is sample weight (kg), and *F_c_* is the correction factor (including recovery factor, attenuation factor, summing in, summing out, growth factor and decay factor) [13,19,25].

### 2.5. Assessment of Environmental Heavy Metal Contamination

This study used three assessment tools to determine the level of contamination of a research area. Pollution indices also involve some heavy metal measurement and geological background data of an area. Geochemical index (I-Geo) aims to determine metal contamination in soil by comparing current concentrations with preindustrial levels. The pollution index (PI) aims to determine metal contamination in soil by comparing current concentration with the regional background. Moreover, ecological risk (RI) and the potential ecological risk of each heavy metal (*E_i_*) are aimed to assess the level of contamination in soil based on the toxicity of heavy metals and environmental response.

#### 2.5.1. Geo-Accumulation Index (I-Geo)

The geo-accumulation index (I-Geo) is a quantitative indicator to measure the degree of soil pollution by heavy metals [26]. I-Geo is expressed by the following equation [27]:

(2) $$I\text{-}\mathrm{Geo}=log2\left(\frac{Cn}{1.5Bn}\right)$$

where *Bn* is the background value in the upper continental crust and *Cn* is the total of heavy metal contents [28], and 1.5 is the background matrix correction factor. The I-Geo was categorized into seven classes: uncontaminated (I-Geo ≤  0), uncontaminated to fairly contaminated (0 < I-Geo ≤ 1), fairly contaminated (1 < I-Geo ≤ 2), fairly contaminated to heavily contaminated (2 < I-Geo ≤ 3), heavily contaminated (3 < I-Geo ≤ 4), heavily contaminated to extraordinarily contaminated (4 < I-Geo ≤ 5), and extraordinarily contaminated (I-Geo ≥ 5) [29,30].

#### 2.5.2. Pollution Index (PI)

The pollution index (PI) of each metal is the ratio of the heavy metal content in the sample to the regional background and calculated according to the following equation:

(3) $$\mathrm{PI}=\frac{Cn}{Bn}$$

where *Cn* is the heavy metal content, *Bn* is the regional background heavy metal content, and PI is the pollution index. The baseline data of regional background defined by Reimann and Caritat [28] are Pb = 14.8 ppm, Zn = 80 ppm, Cu = 75 ppm, and Cd = 0.15 ppm. The PI classified into three classifications as low (PI ≤ 1), middle (1 < PI ≤ 3), or high (PI > 3). The integrated pollution index (IPI) is the mean value for all PI for all considered heavy metals. The IPI are classified as either low (IPI ≤ 1.0), moderate (1.0 < IPI ≤ 2.0), high (2.0 < IPI ≤ 5.0), or extremely high contamination (IPI > 5) [23,30].

#### 2.5.3. Ecological Risk Index (RI)

The ecological risk index (RI) consisted of the sum of individual heavy metals (*E_i_*) and was used to evaluate the potential ecological risk factor of soil pollution by assessing the heavy metals [31]. It represents the biological sensitivity to toxic substances and demonstrates the potential ecological risk caused by heavy metal contamination [32]. The RI was calculated with the following equation:

(4) $$\mathrm{RI}=\sum^{\;}E_{i}\;E_{i}=T_{i}\cdot f_{i}=T_{i}\frac{C_{i}}{B_{i}}$$

where *T_i_* is the toxic response factor for heavy metal, and *f_i_* is the ratio of the measured concentration (*C_i_*) to the background concentration (*B_i_*) of metal in the soil. The *T_i_* values for the heavy metals studied were 5 for Pb and Cu, 1 for Zn, and 30 for Cd. The potential ecological risk of each heavy metal (*E_i_*) was classified into five categories as either low (*E_i_* < 40), fairly (40 ≤ *E_i_* < 80), considerable (80 ≤ *E_i_* < 160), high (160 ≤ *E_i_* < 320), or very high (*E_i_*  > 320). Tang et al. (2013), categorized the RI into four classes: low (RI < 150), moderate (150 < RI ≤ 300), high (300 < RI ≤ 600), and very high (RI > 600) [30].

### 2.6. Statistical Analysis and Map Creation

We used MAPINFO PROFESSIONAL (version 10.5, Precisely, Burlington, MA, USA) to create maps, and ORIGIN PRO 2021 (student version, OriginLab Corp, Northampton, MA, USA) to perform bivariate analysis in order to find significant relationships between the distribution coefficients of radionuclides and heavy metals [19,33]. We conducted a Pearson correlation analysis and calculated the values of Pearson correlation coefficients with a two-tailed significance test (*p*-value at 0.05, 0.01 and 0.001). The equation below expresses the Pearson correlation coefficients:

(5) $$r=\frac{\sum_{i=1}^{n}\left(x_{i}-\bar{x}\right)\left(y_{i}-\bar{y}\right)}{\sqrt{\sum_{i=1}^{n}{\left(x_{i}-\bar{x}\right)}^{2}}\sqrt{\sum_{i=1}^{n}{\left(y_{i}-\bar{y}\right)}^{2}}},$$

where *n* is the sample size, *x_i_* and *y_i_* are the individual sample points indexed, and $\bar{x},\bar{y}$ is the sample mean.

## 3. Results

### 3.1. Radioactivity and Heavy Metal Elements in the Soil Sample

The activity concentration of ^226^Ra, ^232^Th, and ^40^K in surface soil samples from Mamuju is presented in Table 1. The activity concentration of ^226^Ra varied from 232 to 2761 Bq kg^−1^, with an average of 784 Bq kg^−1^. The activity concentration of ^232^Th varied from 424 to 3310 Bq kg^−1^, with an average of 1008 Bq kg^−1^. The activity concentration of ^40^K ranged from 203 to 1655 Bq kg^−1^, with an average 770 Bq kg^−1^.

The concentrations of heavy metal in soil samples from the Mamuju area presented in Table 1 varied from 23 to 145 mg kg^−1^ (Cu), 11 to 293 mg kg^−1^ (Cr), 0.40 to 1.40 mg kg^−1^ (Cd), 109 to 744 mg kg^−1^ (Pb), 4 to 69 mg kg^−1^ (Ni), and 175 to 392 mg kg^−1^ (Zn).

### 3.2. Assessment of Heavy Metal Pollution

The I-Geo, EI, RI, PI, and IPI pollution indices were used to identify heavy metal concentrations of environmental concern in the Mamuju soil samples. These indices numerically describe pollution levels in soils and can be used to calculate the exchangeable soil fraction by representing the real bio-available fraction. The pollution indices of the Mamuju samples are shown in Table 2.

The I-Geo values found for Cu, Cr, Cd, Ni, and Zn show that the samples are within the uncontaminated to fairly contaminated category with an I-geo value of <1, except for the Pb value which has an I-Geo average of 4.11 and a range of 1.48–10. The average I-Geo score for Pb in the Mamuju soil samples is within the heavily contaminated to extraordinary contaminated category (4 < I-Geo ≤ 5).

The PI values, i.e., the ratio of heavy metal content in the samples to the regional background heavy metal content were <1 for Cu, Cr, and Ni, and were therefore within the low contamination category. The PI values of Cd, Pb, and Zn were >3, and were therefore in the high contamination category. The IPI value for the Mamuju soil samples was 2.05 with a range from 1.16 to 3.06, and was therefore within the moderate contamination category.

The EI value is used to evaluate the potential ecological risk of heavy metal soil pollution. EI values for Cu, Cr, Cd, Ni, and Zn in the samples were all <40, and were therefore within the low contamination category. The average EI value for Pb was 102 with a range of 37–251, which is within the medium contamination category (80 ≤ *E_i_* < 160). The RI value is used to evaluate the combined ecological risk of heavy metal soil pollution. The RI values of the samples ranged from 53 to 277, with an average of 125, and were therefore mainly within the low contamination category with some areas included in the moderate contamination category.

## 4. Discussion

In general, Mamuju soil samples were indicated to have a level of environmental radioactivity that is above the worldwide average. Worldwide average activity concentrations of ^40^K, ^226^Ra, and ^232^Th were reported as 412 Bq kg^−1^, 33 Bq kg^−1^, and 45 Bq kg^−1^, respectively [34]. The study result is similar to a previous study conducted by Nugraha et al. (2021), which measured radioactivity in the soil at different sampling points using an HPGe detector. The average activity concentrations of ^238^U, ^232^Th, and ^40^K measured by Nugraha et al. (2021) were 1387 Bq kg^−1^, 1468 Bq kg^−1^, and 301 Bq kg^−1^, respectively [13]. In addition, Nurokhim et al. reported that natural radioactivity in soil samples in the Botteng Utara (Northern Botteng) village had an average concentration of 1042 Bq kg^−1^ for ^226^Ra, 1756 Bq kg^−1^ for ^232^Th, and Bq kg^−1^ for ^40^K [35].

Uranium and thorium are terrestrial naturally occurring radioactive materials (NORMs) which form in the Earth’s crust. ^226^Ra is a decay product of uranium with a half-life of 1600 years. ^226^Ra contained in the soil can potentially accumulate into plants or animals which are consumed by people. Geological conditions strongly influence natural radionuclide activities, and rocks of the alkali basalt group (phonolite, phonotepite, and monolith) found in the Mamuju area (in sub-districts Botteng and Takandeang) are radioactive. Extrusive mafic stone basalt is the most extensive of all frozen rocks, and comprises more than 90% of all volcanic rocks [19]. Moreover, the high concentration of uranium and thorium in the soil, apart from exposing plants, animals, and humans through inhalation and ingestion, can also contribute to gamma radiation exposure. The ambient dose equivalent rate in Mamuju ranged from 235 to 2260 nSv h^−1^. A similar value was reported by Nugraha et al. (2021) with a geometric mean of 613 nSv h^−1^ and a range of 200 to 2300 nSv h^−1^, and Shilfa et al. (2021) reported an ambient dose equivalent rate in Northern Botteng which ranged from 420 to 1430 nSv h^−1^ [14,36].

The heavy metal concentrations in the soil decrease in the sequence Zn > Pb > Cr > Cu > Ni > Cd. The Pb concentration in the Mamuju soil samples ranged from 109 to 744  mg kg^−1^, exceeding the worldwide average of 27  mg kg^−1^ [37]. Moreover, Pb concentrations in the Mamuju soil samples exceeded the thresholds for heavy metals in soil set by the World Health Organization (WHO), Indonesian Ministry of Environment, and the United States of America Environmental Protection Agency (US EPA), which are 0.1 mg kg^−1^, 100 mg kg^−1^, and 200 mg kg^−1^, respectively [37,38,39].

Similar results with heavy metal assessment using pollution indices indicate a possibility that humans may be exposed to these pollutants. The lead value has an I-Geo average of 4.11 and a range of 1.48–10. The average I-Geo value for Pb in the Mamuju soil samples is within the heavily contaminated to extremely contaminated category (4 < I-Geo ≤ 5). The highest values are in Botteng (I-Geo = 6), North Botteng (I-Geo = 7), and Takandeang (I-Geo = 10) areas. The PI values of Cd, Pb, and Zn were >3, and were therefore in the high contamination category. Although the PI values for Cd and Zn are high, these concentrations are still below the limits set by the Indonesian Ministry of Environment. The IPI value for the Mamuju soil samples of 2.05 was, therefore, within the medium contamination category. Moreover, the EI values for Cu, Cr, Cd, Ni, and Zn in the samples were within the low contamination category. However, the average EI value for Pb was 102 with a range of 37–251, which is within the medium contamination category (80  ≤  *E_i_* <  160) and the combined ecological risk of heavy metal soil pollution (RI) values of the samples ranged from 53 to 277, with an average of 125.

This study reveals that Mamuju soil is categorized as moderately contaminated, especially in the case of Pb if the contaminated soil is utilized for agricultural activities. These levels of heavy metal accumulation in soils may increase the phyto-accumulation of these metals in crops grown on the soil. It is really well worth noting that even low levels of heavy metal pollutants can accumulate over time in exposed people and animals [37].

The high concentration of Pb in the soil can affect various things, especially public health. This is because the nature of Pb has high toxic effects on multiple organ problems. Pb can be absorbed into the human body through the respiratory and digestive systems. Exposure to high levels of Pb can cause anemia, kidney weakness, and brain damage. Pb can cross the placental barrier, which means that the exposure of pregnant women to it may also expose their unborn child. Pb can damage the nervous systems of developing babies [40,41]. Based on data from the Central Statistics Agency, Indonesia (2019), upper respiratory tract infections were the most common disease in Mamuju, with 21,070 cases. They are then followed by gastritis, hypertension, and diarrhea with 18,177 cases, 15,876 cases, and 8488 cases, respectively. Based on these data, it is necessary to carry out further thorough research to answer whether the case of Health in Mamuju is closely related to high levels of heavy metals and natural radionuclides. With these facts, Mamuju can be a prospective area for epidemiological studies to be carried out for both low-dose-rate radiation and heavy metal radiation.

Several studies of heavy metals in soils in several regions of the world include Toplica (Serbia), Severodvinsk (Russia), Bayanwula (China), Tong Liao (China), and Kerala (India). [1,4,42,43,44]. Toplica area, Serbia, a tourist city, has heavy metals dominated by arsenic (As; the average concentration 17 mg kg^−1^), Cu, and Ni. Severodvinsk city is the largest industrial center for the navy in north-western Russia with concentrations of As (the average concentration 12.6 mg kg^−1^), Cd, and Pb, which are higher than others. Bayanwula and Tongliao cities in China are uranium mining areas. The Tongliao area has very high Cd, 7–8 times the average soil concentration in China, while the Bayanwula area has a higher concentration of Pb than other heavy metals. In Kerala, India, which is also a high natural background radiation area, the concentration of Pb was found to be higher than in other elements. Table 3 shows the comparison of heavy metal and radionuclide concentrations in the Mamuju area and several regions in the world.

### Correlation of Natural Radionuclides and Heavy Metals

**Figure 2:**
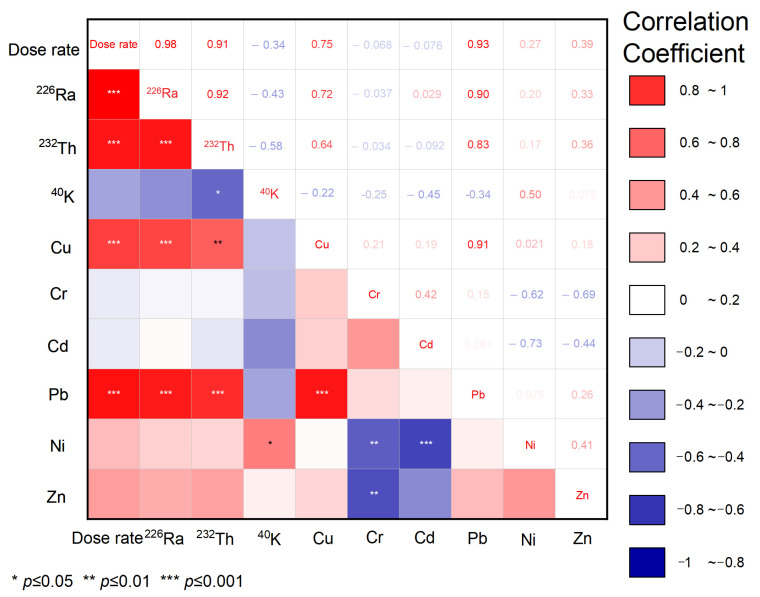
Correlation coefficients between radionuclides and ambient dose equivalent rates (dose rate) and heavy metals.

The correlations between heavy metals and natural radionuclides in soil samples were performed using the ORIGIN PRO 2021 (student version, OriginLab Corp, Northampton, MA, USA). The statistical significance of bivariate analysis is useful to find significant relationships between the distribution coefficients of radionuclides and heavy metals. The correlation coefficient results between radionuclides, ambient dose equivalent rates, and heavy metals in the soil samples are presented in Figure 2. There was a strong positive relationship (*p* ≤ 0.001) between ^226^Ra, ^232^Th, ambient dose equivalent rate, Pb, and Cu with correlation coefficients of 0.92 (^226^Ra and ^232^Th), 0.90 (^226^Ra and Pb), 0.83 (^232^Th and Pb), 0.98 (ambient dose equivalent rate and ^226^Ra), 0.91 (ambient dose equivalent rate and ^232^Th), and 0.91 (Cu and Pb). The strong correlation between ^226^Ra, ^232^Th, and Pb is due to the decay processes of the two radionuclides into Pb isotopes/elements, such as ^214^Pb, ^212^Pb, ^210^Pb, ^208^Pb, and ^206^Pb. The Pb isotope is a decay chain product of uranium (the parent of ^226^Ra) and thorium. ^214^Pb is a radioactive element and has a half-life of 26.8 min, ^210^Pb has a half-life of 22.3 years, and ^206^Pb is the stable isotope (the end product of the uranium decay chain). As for thorium decay, ^212^Pb is a radioactive element with a half-life of 10.6 h, and ^208^Pb is stable, which is the end product of the thorium decay chain [11,19,34,45,46]. All the Pb isotopes have identical chemical properties and toxicity. In addition, the Pb radioisotope (unstable Pb isotope) has tremendous potential for toxicity because it emits beta radiation. ^214^Pb will decay to ^214^Bi, ^212^Pb will decay to ^212^Bi, and ^210^Pb will decay to ^210^Bi. Beta radiation will be hazardous if it enters the human body, either from breathing or digestion [34].

Meanwhile, moderate and even negative correlation coefficients were found between radionuclides and some heavy metals (Cr, Cd, Ni, and Zn). The relationship between ^40^K and the ambient dose equivalent rate was a negative correlation. This negative correlation occurs because the ^226^Ra and ^232^Th contents are high, so the K concentration is not significant.

Lead occurs naturally in the environment. However, anthropogenic activities, such as mining and burning fossil fuels, including transportation, contribute to the release of high concentrations of Pb to the environment. However, there are no mining and industrial activities in Mamuju. The higher correlation coefficient for these elements is due to the fact that the main source of these metals in this area might be due to the parent rock or soil (i.e., a geogenic source) and burning of fossil fuels. Regarding the geogenic sources, the previous study reported rock types in the Mamuju area are classified into alkaline basalt groups, such as gummite (>70% Th), davidite (15% Fe), thorianite (12% Pb), and autunite (>48% U) [20].

## 5. Conclusions

Mamuju is a unique area because it is highly naturally radioactive and has a high concentration of heavy metals. The heavy metal concentrations in the soil decrease in the sequence Zn > Pb > Cr > Cu > Ni > Cd. A strong positive correlation was found between ^226^Ra, ^232^Th, and Pb. The strong correlation between ^226^Ra, ^232^Th, and Pb is due to the decay processes of the two radionuclides into Pb isotopes/elements, such as ^214^Pb, ^212^Pb, ^210^Pb, ^208^Pb, and ^206^Pb. The Pb isotope is a decay chain product of uranium (the parent of ^226^Ra) and thorium. The ecological risk index (RI) and cumulative pollution index (IPI) values in Mamuju are 2.05 and 125, respectively, and are therefore within the moderately contaminated category. Lead concentrations in the Mamuju soil samples exceeded the thresholds for heavy metals in soil set by the World Health Organization (WHO), the Indonesian Ministry of Environment, and the United States of America Environmental Protection Agency (US EPA). Moreover, Pb concentration in the Mamuju soil samples ranged from 109 to 744 mg kg^−1^, exceeding the worldwide average of 27 mg kg^−1^. It is possible that these factors might cause negative health effects among the residents of the area.

The determination of the heavy metal in a soil sample is a fundamental study to the identification, monitoring, and assessment of potential sources of pollution in the area. This study is expected to assist local governments, central government, and various stakeholders in making policies regarding environmental safety and population health. This study did not conduct heavy metal measurements in foodstuffs and drinking water. Therefore, we suggest that it is necessary to carry out continuous research, especially the measurement of heavy metals in food, water, and humans (bioassays), to obtain more comprehensive data. With these facts, Mamuju can be a prospective area for epidemiological studies to be carried out for both low-dose-rate radiation and heavy metal radiation.

## Figures and Tables

**Table 1:** The descriptive statistics of ambient dose equivalent rate, natural radionuclides, and heavy metals in a soil sample from Mamuju.

Measurement	Average	Min	Max	SD	Skewness	Kurtosis
Ambient dose equivalent rate (nSv h^−1^)	699 ± 65	235 ± 20	2260 ± 219	634	1.70	2.12
^226^Ra (Bq kg^−1^)	784 ± 40	232 ± 12	2761 ± 138	707	2.01	3.57
^232^Th (Bq kg−^1^)	1008 ± 45	424 ± 22	3310 ± 166	954	1.80	1.74
^40^K (Bq kg^−1^)	770 ± 46	203 ± 16	1655 ± 99	506	0.36	−1.25
Cu (mg kg^−1^)	71 ± 4	23 ± 1	145 ± 6	44	0.52	−1.53
Cr (mg kg^−1^)	84 ± 4	11 ± 0.55	293 ± 12	72	2.17	4.69
Cd (mg kg^−1^)	0.69 ± 0.14	0.40 ± 0.14	1.40 ± 0.14	0.35	1.45	0.91
Pb (mg kg^−1^)	303 ± 18	109 ± 7	744 ± 37	204	0.98	−0.27
Ni (mg kg^−1^)	41 ± 2	4 ± 1.50	69 ± 3	20	−0.68	−0.39
Zn (mg kg^−1^)	304 ± 15	175 ± 9	392 ± 20	62	−0.77	0.12

**Table 2:** Heavy metal pollution indices in Mamuju.

Pollution Indices	Descriptive Statistic	Heavy Metal					
		Cu	Cr	Cd	Pb	Ni	Zn
I-Geo	Average	0.19	0.17	0.92	4.11	0.11	0.76
	min	0.06	0.02	0.54	1.48	0.01	0.44
	max	0.39	0.59	1.87	10	0.18	0.98
	SD	0.12	0.14	0.47	2.76	0.05	0.15
	Skewness	0.52	2.17	1.45	0.98	−0.68	−0.77
	Kurtosis	−1.53	4.69	0.91	−0.27	−0.39	0.12
EI	Average	4.76	1.68	9.19	102	2.71	3.80
	min	1.53	0.22	5.33	37	0.27	2.19
	max	9.67	5.86	19	251	4.60	4.90
	SD	2.97	1.44	4.66	69	1.31	0.77
	Skewness	0.52	2.17	1.45	0.98	−0.68	−0.77
	Kurtosis	−1.53	4.69	0.91	−0.27	−0.39	0.12
PI	Average	0.95	0.84	4.59	20	0.54	3.80
	min	0.31	0.11	2.67	7.36	0.05	2.19
	max	1.93	2.93	9.33	50	0.92	4.90
	SD	0.59	0.72	2.33	14	0.26	0.77
	Skewness	0.52	2.17	1.45	0.98	−0.68	−0.77
	Kurtosis	−1.53	4.69	0.91	−0.27	−0.39	0.12
RI	Average	125					
	min	53					
	max	277					
	SD	72					
	Skewness	0.89					
	Kurtosis	−0.46					
IPI	Average	2.05					
	min	1.16					
	max	3.06					
	SD	0.66					
	Skewness	0.49					
	Kurtosis	−1.39					

**Table 3:** The comparison of heavy metal and radionuclide concentrations in the Mamuju area and several regions in the world.

Area	^226^Ra (Bq kg^−1^)	^232^Th (Bq kg^−1^)	^40^K (Bq kg^−1^)	Cu (mg kg^−1^)	Cr (mg kg^−1^)	Cd (mg kg^−1^)	Pb (mg kg^−1^)	Ni (mg kg^−1^)	Zn (mg kg^−1^)	Reference
This Study	784	1008	770	71	84	0.69	303	41	304	This study
Top Lica, Serbia	30	37	492	40	100	0.12	47	118	111	[1]
Severodvinsk, Rusia	12	9	191	8	20	0.19	10	10	25	[44]
Bayanwula, China	25	29	923	5	15	0.01	74	5	118	[43]
Tong liao, China	13	16	747	8	-	1.73	35	-	30	[42]
Kerala, India	350	2825	180	-	116	0.26	83	36		[4,14]
Soil World Average	32	45	400	20	100	1.00	10	40	50	[34,39]

## Data Availability

Data set available on request to corresponding authors.
